# Supplementary material for: Optimal Postoperative Surveillance Strategies for Colorectal Cancer: A Retrospective Observational Study
Source: Cancers (Basel). 2021 Jul 13;13(14):3502. doi: 10.3390/cancers13143502 (PMC8306168; doi:10.3390/cancers13143502)
Supplement: Supplementary file 1 [file cancers-13-03502-s001.zip › cancers-1212668-supplementary.pdf]

cancers

MDPI

Article

Optimal postoperative surveillance strategies for colorectal cancer: a retrospective observational study

Min Young Park; In Ja Park; Hyo Seon Ryu; Jay Jung; Minsung Kim; Seok-Byung Lim; Chang Sik Yu; Jin Cheon Kim

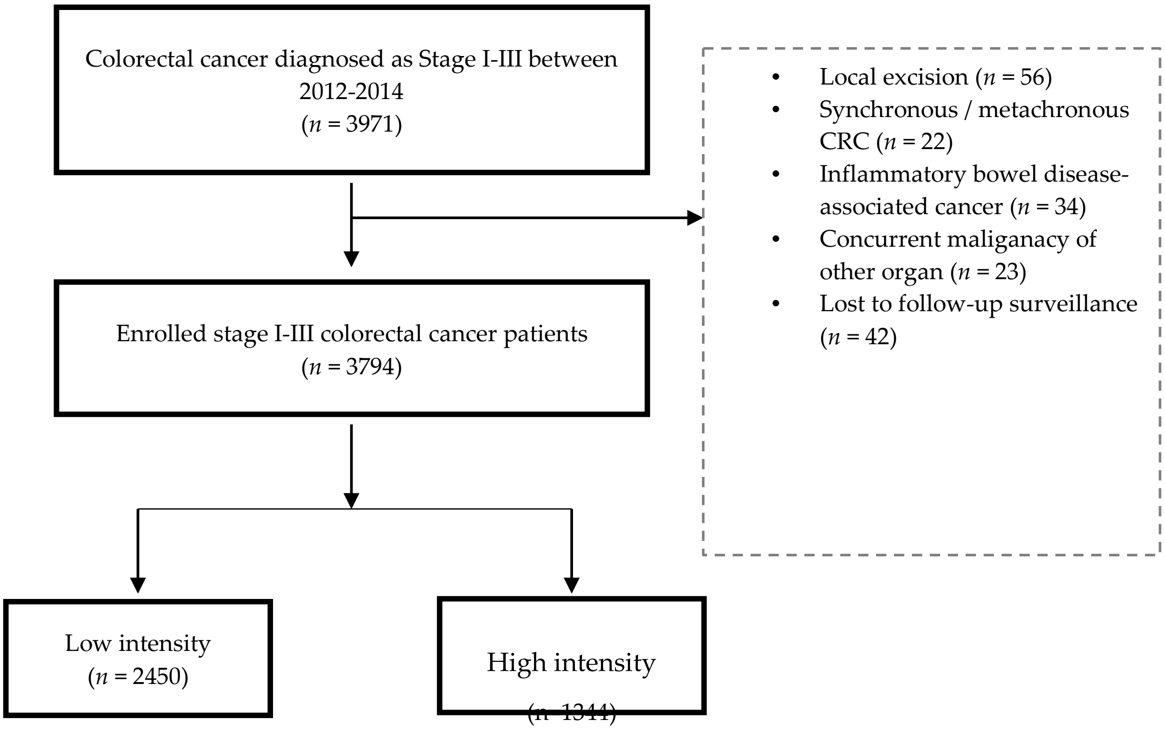

Figure S1. Flowchart of included patients.

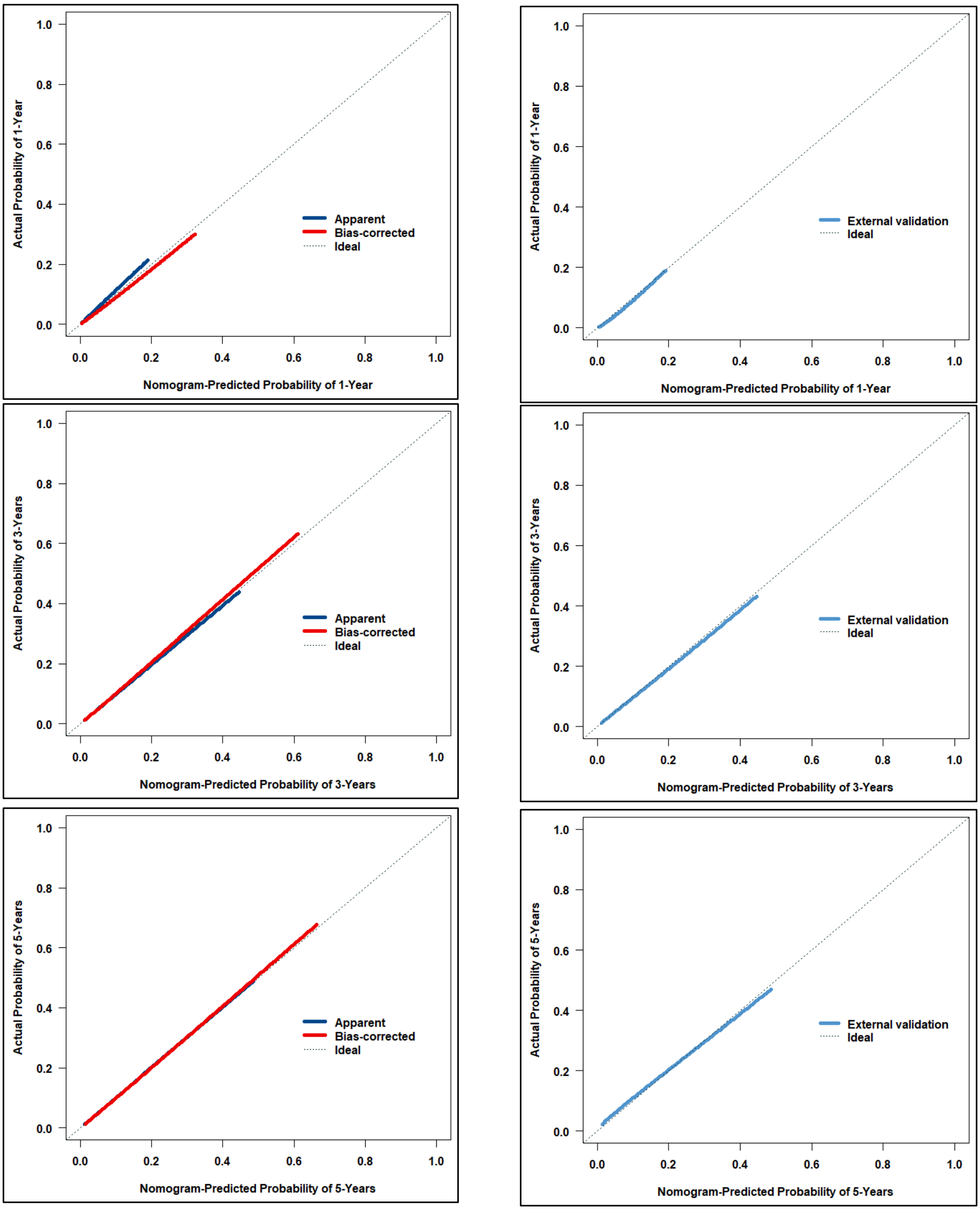

Figure S2. Calibration plots in the (A) internal and (B) external validation cohorts.

Table S1. Subgroup analysis according to the stage.

Variables	Surveillance intensity		*P*-value
	**Lower intensity**	**Higher intensity**	
**Stage I (*N* = 1131)**	***N* = 953**	***N* = 178**	
Overall recurrence, No (%)	24 (2.5)	18 (10.1)	<0.001
Overall survival, months, mean (SD)	59.07 (18.33)	60.15 (18.59)	0.474
Recurrence free survival, months, mean (SD)	57.38 (19.32)	55.34 (21.37)	0.203
Post recurrence survival, months, mean (SD)	31.83 (27.79)	40.44 (23.72)	0.286
**Stage II (*N* = 1277)**	***N* = 818**	***N* = 459**	
Overall recurrence, No (%)	56 (6.8)	69 (15.0)	<0.001
Overall survival, months, mean (SD)	55.73 (21.57)	58.18 (21.73)	0.054
Recurrence free survival, months, mean (SD)	52.06 (23.40)	50.57 (25.04)	0.288
Post recurrence survival, months, mean (SD)	32.73 (22.96)	41.19 (21.54)	0.037
**Stage III (*N* = 1187)**	***N* = 535**	***N* = 652**	
Overall recurrence, No (%)	95 (17.8)	172 (26.4)	<0.001
Overall survival, months, mean (SD)	53.86 (24.77)	26.79 (21.71)	0.030
Recurrence free survival, months, mean (SD)	47.69 (27.38)	47.83 (25.17)	0.931
Post recurrence survival, months, mean (SD)	27.16 (22.44)	31.78 (22.80)	0.110

No, number; SD, standard deviation

Table S2. Factors affecting recurrence-free survival according to the recurred site.

Factors	Univariate		Multivariate	
	HR (95% CI)	*P*	HR (95% CI)	*P*
**Intra-abdominal recurrence**				
Age (years)	1.006 (0.996–1.017)	0.232		
Sex	0.835 (0.660–1.058)	0.135	0.804 (0.634–1.019)	0.071
APCT intensity	4.685 (3.719–5.904)	<0.001	2.527 (1.973–3.236)	<0.001
Differentiation				
WD/MD	Ref		Ref	
PD/SRC/MUC	2.395 (1.722–3.331)	<0.001	1.643 (1.174–2.298)	0.004
(y)pT stage				
0–2	Ref		Ref	
3–4	5.925 (4.145–8.471)	<0.001	2.794 (1.907–4.093)	<0.001
(y)pN stage				
0	Ref		Ref	
1	3.212 (2.468–4.179)	<0.001	1.678 (1.262–2.230)	<0.001
2	7.957 (5.918–10.699)	<0.001	2.955 (2.106–4.145)	<0.001
Lymphovascular invasion	2.534 (2.017–3.184)	<0.001	1.094 (0.846–1.415)	0.491
Perineural invasion	3.431 (2.704–4.354)	<0.001	1.614 (1.249–2.086)	<0.001
Resection margin	4.661 (2.927–7.423)	<0.001	2.147 (1.339–3.443)	0.002
**Intra-thoracic recurrence**				
Age (years)	1.008 (0.995–1.021)	0.238		
CCT intensity	2.496 (1.802–3.459)	<0.001	2.066 (1.488–2.868)	<0.001
Differentiation				
WD/MD	Ref			
PD/SRC/MUC	0.758 (0.388–1.480)	0.417		
(y)pT stage				
0–2	Ref		Ref	
3–4	3.479 (2.399–5.046)	<0.001	1.915 (1.279–2.868)	0.002
(y)pN stage				
0	Ref		Ref	
1	2.907 (2.128–3.972)	<0.001	1.950 (1.395–2.724)	<0.001
2	4.205 (2.751–6.429)	<0.001	2.225 (1.393–3.555)	0.001
Lymphovascular invasion	1.841 (1.373–2.471)	<0.001	0.935 (0.674–1.297)	0.687
Perineural invasion	3.593 (2.667–4.841)	<0.001	2.222 (1.600–3.087)	<0.001
Resection margin	4.271 (2.321–7.857)	<0.001	2.422 (1.306–4.493)	0.005

HR, hazard ratio; CI, confidence interval; WD, well differentiated; MD, moderately differentiated; PD, poorly differentiated; SRC, signet ring cell type; MUC, mucinous carcinoma; Ref, reference; APCT, abdomino-pelvic computed tomography; CCT, chest computed tomography.

Table S3. Factors affecting post-recurrence survival according to the recurred site.

Factors	Univariate		Multivariate	
	HR (95% CI)	*P*	HR (95% CI)	*P*
**Intra-abdominal recurrence**				
Age (years)	1.029 (1.016–1.043)	<0.001	1.028 (1.015–1.041)	<0.001
Sex	1.025 (0.761–1.380)	0.873		
APCT intensity	1.035 (0.768–1.393)	0.823		
Differentiation				
WD/MD	Ref		Ref	
PD/SRC/MUC	2.922 (2.012–4.244)	<0.001	2.367 (1.594–3.515)	<0.001
(y)pT stage				
0–2	Ref			
3–4	1.314 (0.807–2.140)	0.273		
(y)pN stage				
0	Ref		Ref	
1	1.209 (0.859–1.703)	0.277	1.309 (0.917–1.867)	0.138
2	1.613 (1.116–2.329)	0.011	1.343 (0.893–2.021)	0.156
Lymphovascular invasion	1.556 (1.164–2.079)	0.003	1.097 (0.797–1.510)	0.570
Perineural invasion	1.240 (0.918–1.675)	0.161	1.110 (0.805–1.531)	0.525
Resection margin	1.238 (0.718–2.137)	0.442		
Curative resection	0.202 (0.140–0.290)	<0.001	0.233 (0.161–0.338)	<0.001
**Intra-thoracic recurrence**				
Age (years)	1.019 (1.002–1.036)	0.028	1.030 (1.012–1.048)	0.001
Sex	1.015 (0.692–1.489)	0.938		
CCT intensity	0.848 (0.545–1.319)	0.464		
Differentiation				
WD/MD	Ref		Ref	
PD/SRC/MUC	6.894 (3.010–15.792)	<0.001	2.832 (1.174–6.833)	0.021
(y)pT stage				
0–2	Ref			
3–4	1.171 (0.720–1.905)	0.525		
(y)pN stage				
0	Ref		Ref	
1	1.330 (0.872–2.028)	0.185	1.403 (0.866–2.272)	0.169
2	2.288 (1.355–3.864)	0.002	2.417 (1.246–4.690)	0.009
Lymphovascular invasion	1.525 (1.039–2.238)	0.031	1.071 (0.670–1.712)	0.775
Perineural invasion	0.829 (0.558–1.230)	0.351		
Resection margin	1.966 (0.953–4.056)	0.067	1.539 (0.709–3.342)	0.275
Curative resection	0.203 (0.130–0.316)	<0.001	0.207 (0.132–0.327)	<0.001

HR, hazard ratio; CI, confidence interval; Ref, reference; WD, well differentiated; MD, moderately differentiated; PD, poorly differentiated; SRC, signet ring cell type; MUC, mucinous carcinoma.

Table S4. Clinical characteristics of patients in the development and validation cohorts.

Variables	Development (n = 3794)	Validation (n = 2215)	P
Age, mean (SD)	61.3 (11.6)	60.8 (10.8)	0.107
Gender, No (%)			0.038
Male	2221 (58.5)	1357 (61.3)	
Female	1573 (41.5)	858 (38.7)	
Differentiation, No (%)			0.846
WD/MD	3539 (93.3)	2069 (93.4)	
PD/SRC/MUC	255 (6.7)	146 (6.6)	
(y)pT, No (%)			<0.001
0	203 (5.4)	55 (2.5)	
1	689 (18.2)	352 (15.9)	
2	651 (17.2)	346 (15.6)	
3	1970 (51.9)	1352 (61.0)	
4	281 (7.4)	110 (5.0)	
(y)pN, No (%)			<0.001
0	2597 (68.5)	1449 (65.4)	
1	930 (24.5)	540 (24.4)	
2	267 (7.0)	226 (10.2)	
Perineural invasion, No (%)	570 (15.3)	377 (17.0)	0.072
Lymphovascular invasion, No (%)	1016 (27.2)	449 (20.3)	<0.001

SD, standard deviation; WD, well differentiated; MD, moderately differentiated; PD, poorly differentiated; SRC, signet ring cell type; MUC, mucinous carcinoma.

Table S5. Predictors of recurrence after radical resection.

Factors	Univariate		Multivariate	
	**HR (95% CI)**	***P***	**HR (95% CI)**	***P***
Age (per 10 years increase)	1.06 (0.98–1.15)	0.135		
Gender				
Male	Ref		Ref	
Female	0.82 (0.67–0.99)	0.040	0.82 (0.67–0.99)	0.037
Differentiation				
WD/MD	Ref			
PD/SRC/MUC	1.34 (0.99–1.82)	0.059		
(y)pT stage				
0	Ref		Ref	
1	0.32 (0.13–0.76)	0.010	0.32 (0.13–0.76)	0.010
2	1.19 (0.58–2.44)	0.638	1.20 (0.58–2.45)	0.626
3	1.95 (0.99–3.83)	0.053	1.98 (1.01–3.90)	0.047
4	3.54 (1.74–7.21)	<0.001	3.67 (1.81–7.45)	<0.001
(y)pN stage				
0	Ref		Ref	
1	2.08 (1.67–2.61)	<0.001	2.06 (1.66–2.57)	<0.001
2	3.48 (2.63–4.61)	<0.001	3.50 (2.68–4.56)	<0.001
Lymphovascular invasion	0.98 (0.79–1.21)	0.854		
Perineural invasion	1.70 (1.37–2.11)	<0.001	1.66 (1.34–2.05)	<0.001

HR, hazard ratio; CI, confidence interval; Ref, reference; WD, well differentiated; MD, moderately differentiated; PD, poorly differentiated; SRC, signet ring cell type; MUC, mucinous carcinoma.

Table S6. Demographic and clinical characteristics of patients with high recurrence risk (N=1228).

Variables	Surveillance Intensity: Lower Intensity (*n* = 530)	Surveillance Intensity: Higher Intensity (*n* = 698)	*P*-value
Age, mean (IQR)	63.3 (55.8–72.0)	59.7 (52.0–69.0)	<0.001
Gender, No (%)			0.345
Male	315 (59.4)	434 (62.2)	
Female	215 (40.6)	264 (37.8)	
Cancer site, No (%)			0.591
Colon	340 (64.2)	437 (62.6)	
Rectum	190 (35.8)	261 (37.4)	
Differentiation, No (%)			0.047
WD/MD	486 (91.7)	615 (88.1)	
PD/SRC/MUC	44 (8.3)	83 (11.9)	
Total lymph nodes, No (%)			0.840
<12	10 (1.9)	15 (2.1)	
≥12	520 (98.1)	683 (97.9)	
(y)pT, No (%)			0.736
2	7 (1.3)	9 (1.3)	
3	407 (76.8)	523 (74.9)	
4	116 (21.9)	166 (23.8)	
(y)pN, No (%)			0.052
0	108 (20.4)	119 (17.0)	
1	324 (61.1)	413 (59.2)	
2	98 (18.5)	166 (23.8)	
Perineural invasion, No (%)	194 (36.6)	268 (38.4)	0.552
Lymphovascular invasion, No (%)	263 (49.6)	357 (51.1)	0.604
Resection margin, No (%)	17 (3.2)	28 (4.0)	0.540
Total imaging studies, mean (SD)	2.31 (0.85)	4.44 (7.27)	<0.001
Overall recurrence, No (%)	102 (19.2)	193 (27.7)	0.001
Local recurrence, No (%)	7 (1.3)	20 (2.9)	0.078
Systemic recurrence, No (%)	95 (17.9)	173 (24.8)	0.004

IQR, inter-quartile range; No, number; WD, well differentiated; MD, moderately differentiated; PD, poorly differentiated; SRC, signet ring cell type; MUC, mucinous carcinoma; SD, standard deviation
